# Unraveling the genetic basis of grain number-related traits in a wheat-*Agropyron cristatum* introgressed line through high-resolution linkage mapping

**DOI:** 10.1186/s12870-023-04547-7

**Published:** 2023-11-15

**Authors:** Yun-Feng Xu, Fei-Fei Ma, Jin-Peng Zhang, Hong Liu, Li-Hui Li, Diao-Guo An

**Affiliations:** 1 Center for Agricultural Resources Research, Institute of Genetics and Developmental Biology, Chinese Academy of Sciences, Shijiazhuang, 050021 China; 2 The National Key Facility for Crop Gene Resources and Genetic Improvement, Institute of Crop Science, Chinese Academy of Agricultural Sciences, Beijing, 100081 China

**Keywords:** Grain number per spike, Grain number per spikelet, Quantitative trait locus (QTL), High-density linkage map, Wheat-*Agropyron cristatum* germplasm

## Abstract

**Background:**

Grain number per spike (GNS) is a pivotal determinant of grain yield in wheat. Pubing 3228 (PB3228), a wheat-*Agropyron cristatum* germplasm, exhibits a notably higher GNS.

**Results:**

In this study, we developed a recombinant inbred line (RIL) population derived from PB3228/Gao8901 (PG-RIL) and constructed a high-density genetic map comprising 101,136 loci, spanning 4357.3 cM using the Wheat 660 K SNP array. The genetic map demonstrated high collinearity with the wheat assembly IWGSC RefSeq v1.0. Traits related to grain number and spikelet number per spike were evaluated in seven environments for quantitative trait locus (QTL) analysis. Five environmentally stable QTLs were detected in at least three environments. Among these, two major QTLs, *QGns-4A.2* and *QGns-1A.1*, associated with GNS, exhibited positive alleles contributed by PB3228. Further, the conditional QTL analysis revealed a predominant contribution of PB3228 to the GNS QTLs, with both grain number per spikelet (GNSL) and spikelet number per spike (SNS) contributing to the overall GNS trait. Four kompetitive allele-specific PCR (KASP) markers that linked to *QGns-4A.2* and *QGns-1A.1* were developed and found to be effective in verifying the QTL effect within a diversity panel. Compared to previous studies, *QGns-4A.2* exhibited stability across different trials, while *QGns-1A.1* represents a novel QTL. The results from unconditional and conditional QTL analyses are valuable for dissecting the genetic contribution of the component traits to GNS at the individual QTL level and for understanding the genetic basis of the superior grain number character in PB3228. The KASP markers can be utilized in marker-assisted selection for enhancing GNS.

**Conclusions:**

Five environmentally stable QTLs related to grain number and spikelet number per spike were identified. PB3228 contributed to the majority of the QTLs associated with GNS.

**Supplementary Information:**

The online version contains supplementary material available at 10.1186/s12870-023-04547-7.

## Background

Grain number per spike (GNS) is one of three component traits that contribute to the grain yield of wheat (*Triticum aestivum* L.) [1]. Spikelet number per spike (SNS), which includes the total number of spikelet as well as fertile and sterile spikelet (referred to as TSS, FSS, and SSS, respectively), and grain number of spikelet (GNSL) are the two direct components that show a positive correlation with GNS [2–5]. Selecting wheat varieties with larger spike styles, containing more spikelets, has proven to be an efficient strategy for increasing yield potential [6, 7].

Classical breeding has revealed that GNS, along with its related traits such as GNSL and SNS, are quantitative traits that are controlled by multiple quantitative trait loci (QTLs) [8–10]. Until now, QTLs for GNS and its related traits have been detected through linkage mapping in many studies and distributed across nearly all of the 21 chromosomes of wheat [11–14]. Stable-expressed QTLs for GNS and TSS, which have shown effectiveness across various environments and genetic backgrounds have been identified on chromosomes 1A, 1B, 2D, 3A, 4B, 5A and 7A [4, 8, 12, 13, 15–20].

Several genes related to FSS or GNSL have been reported. For instance, *WAPO1*, an ortholog of the rice gene *APO1*, is located on chromosome 7AL and can affect spikelet number [15, 17]. The gene *GNI1* encods a HD-Zip I transcription factor and the reduced-function allele of *GNI-A1* can increase the GNSL [21]. Additionally, *TaMOC1* [22] and *TaDEP1* [23] can affect SNS. *Q* [24] and miR172 [25] are related to GNSL. To identify more genes for GNS, it is essential to detect major QTLs that are mapped by a high-density genetic map with a small confidence interval.

The hexaploidy nature, large genome size, and high percentage of repetitive regions make it difficult to obtain a sufficient number of polymorphic markers in wheat. This results in a deficiency of resolution and coverage for many PCR-based genetic maps of wheat. In recent years, new sequencing technologies have facilitated the development of single nucleotide polymorphism (SNP) arrays. The SNP-based high-density genetic maps, along with the release of the whole genome of the common wheat variety Chinese Spring [26], have facilitated the comparison of QTLs in different researches. For example, Zhai et al. [13] identified a multi-environmentally-stable QTL for SNS on chromosome 7A in Jing 411. In one of our previous research, we discovered a stable locus for SNS within the same interval, which was also conferred by Jing 411 [8]. Subsequent remapping efforts led to the identification of tightly-linked SNPs associated with this locus, utilizing a high-density genetic map based on the Wheat 660 K SNP array. By aligning the SNPs from various studies to the wheat reference genome, we identified an overlapping physical interval.

Previous studies have demonstrated that conditional QTL mapping can effectively reflect the genetic effects of complex traits. In wheat, both unconditional and conditional QTL analyses have been conducted to uncover the dynamic genetic factors that influence target traits. For example, Liu et al. [27] conducted a conditional QTL analysis, revealing that the stable QTLs for grain weight were primarily influenced by grain width. Zhang et al. [28] performed unconditional and conditional QTL mapping, examining the temporal and spatial expression patterns of the stable QTL related to plant height. These findings indicate that conditional analysis is valuable for investigating the developmental behavior of quantitative traits at the QTL level.

The wheat germplasm “Pubing 3228 (PB3228)” was derived from the F_7_ progeny of the cross between *Triticum aestivum* cv. “Fukohokomugi” and *Agropyron cristatum* “Z559” with 21 pairs of chromosomes. It exhibits superior characteristics in yield-related traits, particularly in spike morphological traits, such as large spike and higher grain number [3, 29]. In the present study, a recombinant inbred line (RIL) population derived from the cross between “PB3228” and “Gao 8901 (G8901)” (PG-RIL) was utilized to achieve the following objectives: (i) construct a high-density genetic linkage map; (ii) identify stable QTLs for GNS and elucidate the genetic contribution of the individual component traits to GNS at the specific QTL level; (iii) dissect the genetic basis of the superior grain number characteristic exhibited by PB3228, and predict potential candidate genes associated with the key QTLs; and (iv) develop user-friendly markers for application in marker-assisted selection (MAS) during the breeding process. The results of this study could potentially provide beneficial QTLs for use in MAS and fine-mapping, thereby aiding in the enhancement of yield in wheat breeding and offering further insights into the underlying genetic mechanisms governing superior grain number in wheat.

## Results

### Phenotypic variation and correlations among traits

The broad-sense heritability (*h*_*B*_^*2*^) of the spikelet number traits (67.9% and 59.8% for TSS and FSS, respectively) were higher than grain number-related traits (50.9% and 53.8% for GNS and GNSL, respectively. Table S1). Significant genetic and environmental effects were observed for all the investigated traits (Table S1). These findings underscore the significance of genetic background in explaining the overall phenotypic variation of the four traits, while also indicating that environmental factors exert a greater influence on GNS and GNSL as opposed to TSS and FSS. PB3228 demonstrated significantly higher phenotypic values for all the traits and environments, with the exception of TSS in 2016LC (Table S1). The phenotypic values of the four traits exhibited broad and continuous variation among the 176 RILs, displaying significant transgressive segregation for both the parental lines (Table S1, Fig. S2). This phenomenon could potentially be attributed to the distinct genetic background of the two parents and the complex polygenic inheritance patterns governing these traits. The correlation analysis indicated a significant relationship between GNS and all three examined traits. Specifically, GNSL exhibited a stronger correlation with GNS (*r* = 0.79, *P* < 0.001), while TSS (*r* = 0.55, *P* < 0.001) and FSS (*r* = 0.68, *P* < 0.001) displayed comparatively lower correlations GNS (Table 1). Additionally, GNSL displayed positive correlations with both TSS and FSS. These findings suggest that within the PG-RIL genetic background, GNSL potentially exerts a more significant influence on GNS compared to the influence of TSS/FSS.

**Table 1:** Correlation coefficients between the investigated traits of the RILs and their parents

Traits	Environments	GNS	GNSL	TSS
GNSL	Across	0.7874^***^		
	2015LC	0.8470^***^		
	2016LC	0.7682^***^		
TSS	Across	0.5532^***^	0.3304^***^	
	2013LC	0.4640^***^	NA	
	2014LC	0.4454^***^	NA	
	2015LC	0.4960^***^	0.2787^***^	
	2015SJZ	0.4037^***^	NA	
	2016LC	0.4229^***^	0.1999^**^	
FSS	Across	0.6781^***^	0.4216^***^	0.9505^***^
	2013LC	0.6213^***^	NA	0.9211^***^
	2014LC	0.6276^***^	NA	0.9110^***^
	2015LC	0.6570^***^	0.4429^***^	0.9247^***^
	2015SJZ	0.4636^***^	NA	0.9392^***^
	2016LC	0.6485^***^	0.3330^***^	0.8907^***^

*GNS* grain number per spike, *GNSL* grain number per spikelet, *TSS* total spikelet number per spike, *FSS* fertile spikelet number per spike, *NA* not applicated

^**^p ≤ 0.01

^***^p ≤ 0.001

### Genotyping and map construction

The 176 RILs of PG-RIL population along with their parents were genotyped using the SNP probes derived from the Wheat 660 K SNP array. The RILs were determined to have Call Rates ranged from 97.0% to 98.8%, Heterozygote Rate ranged from 2.6% to 7.9%; and all were passed the quality control. Among the 630,517 SNP probes, 115,836 that had Call Rate > 97%, Heterozygote Rate < 20%, and Minor Allele Frequency > 5% were selected for further analysis. Among them, 101,151 that had Conversion Type “Poly High Resolution (PHR)” and showed polymorphism between the two parents PB3228 and G8901 were selected for map construction. The 101,151 functional SNPs were binned to 4477 bins. The representative SNPs of the 4,477 bins along with 45 other markers were used for linkage analysis and map construction.

**Fig. 1:**
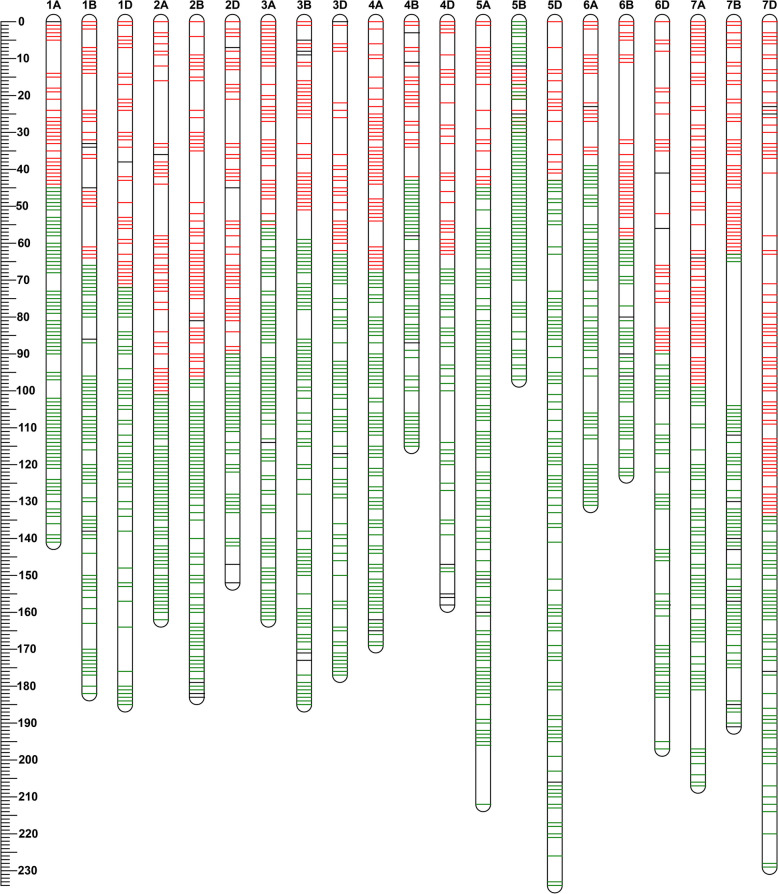
The high-density genetic linkage map of recombinant inbred line (RIL) derived from PB3228/Gao8901 (PG-RIL) population. The ruler on the left represents the position of the marker on the chromosome. The markers with red and green color are from the short and long arms, respectively, of the wheat whole genome assembly IWGSC RefSeq v1.0; while the markers with black color have no physical position information

In total, a high-density genetic map with 101,136 loci spanning 4357.3 cM was released based on 101,091 SNP markers and 45 SSR, kompetitive allele-specific PCR (KASP), or morphological markers (Table 2). Most markers were mapped to the A genome (49.0%) and B genome (41.8%), whereas only 9.2% markers were mapped to D genome. For the map lengths, A, B, and D genomes covered 32.9%, 30.2%, and 36.9% of the total map length, respectively. The chromosome sizes ranged from 134.25 cM (chromosome 6A) to 311.75 cM (chromosome 7D), with an average of 207.49 cM per chromosome. The number of markers on each chromosome ranged from 261 (chromosome 4D) to 10,879 (chromosome 1A), with a mean of 4,816 markers per chromosome. The average distance between adjacent bin markers ranged from 0.67 cM for 3A to 1.64 cM for 6D, with an average of 0.96 cM. The maximum distance between adjacent bin markers ranged from 6.25 cM for 3A to 29.57 cM for 3B. Gaps exceeding 20 cM were present in chromosomes 3A (24.9 cM), 2B (21.98 cM), 3D (28.65 cM), 6B (22.69 cM) and 7B (29.57 cM) (Fig. 1).

**Table 2:** Summary information of the recombinant inbred line population derived from PB3228/Gao8901 (PG-RIL) high-resolution genetic map

Chromosomes	Locus number	Marker number	Map length(cM)	Average distance(cM)	Max distance(cM)
1A	240	10879	210.24	0.88	16.33
1B	244	4032	202.91	0.83	16.90
1D	162	2233	208.49	1.29	17.33
2A	255	6678	217.99	0.85	15.06
2B	274	8456	228.88	0.84	21.98
2D	149	1593	215.62	1.45	16.19
3A	285	4903	190.77	0.67	6.25
3B	243	6363	212.40	0.87	11.84
3D	157	1203	232.05	1.48	28.65
4A	278	8004	194.56	0.70	7.97
4B	180	3604	138.43	0.77	11.47
4D	117	261	174.52	1.49	15.90
5A	295	7126	261.19	0.89	16.62
5B	287	10522	220.34	0.77	10.43
5D	162	1310	255.38	1.58	12.87
6A	176	6856	134.25	0.76	10.29
6B	165	5192	145.07	0.88	22.69
6D	127	695	208.76	1.64	12.21
7A	312	5116	224.11	0.72	18.20
7B	197	4109	169.63	0.86	29.57
7D	211	2001	311.75	1.48	14.46
Group1	646	17144	621.64	0.96	17.33
Group2	678	16727	662.49	0.98	21.98
Group3	685	12469	635.22	0.93	28.65
Group4	575	11869	507.51	0.88	15.90
Group5	744	18958	736.91	0.99	16.62
Group6	468	12743	488.08	1.04	22.69
Group7	720	11226	705.49	0.98	29.57
GenomeA	1841	49562	1433.11	0.78	18.20
GenomeB	1590	42278	1317.66	0.83	29.57
GenomeD	1085	9296	1606.57	1.48	28.65
Total	4516	101136	4357.34	0.96	29.57

**Fig. 2:**
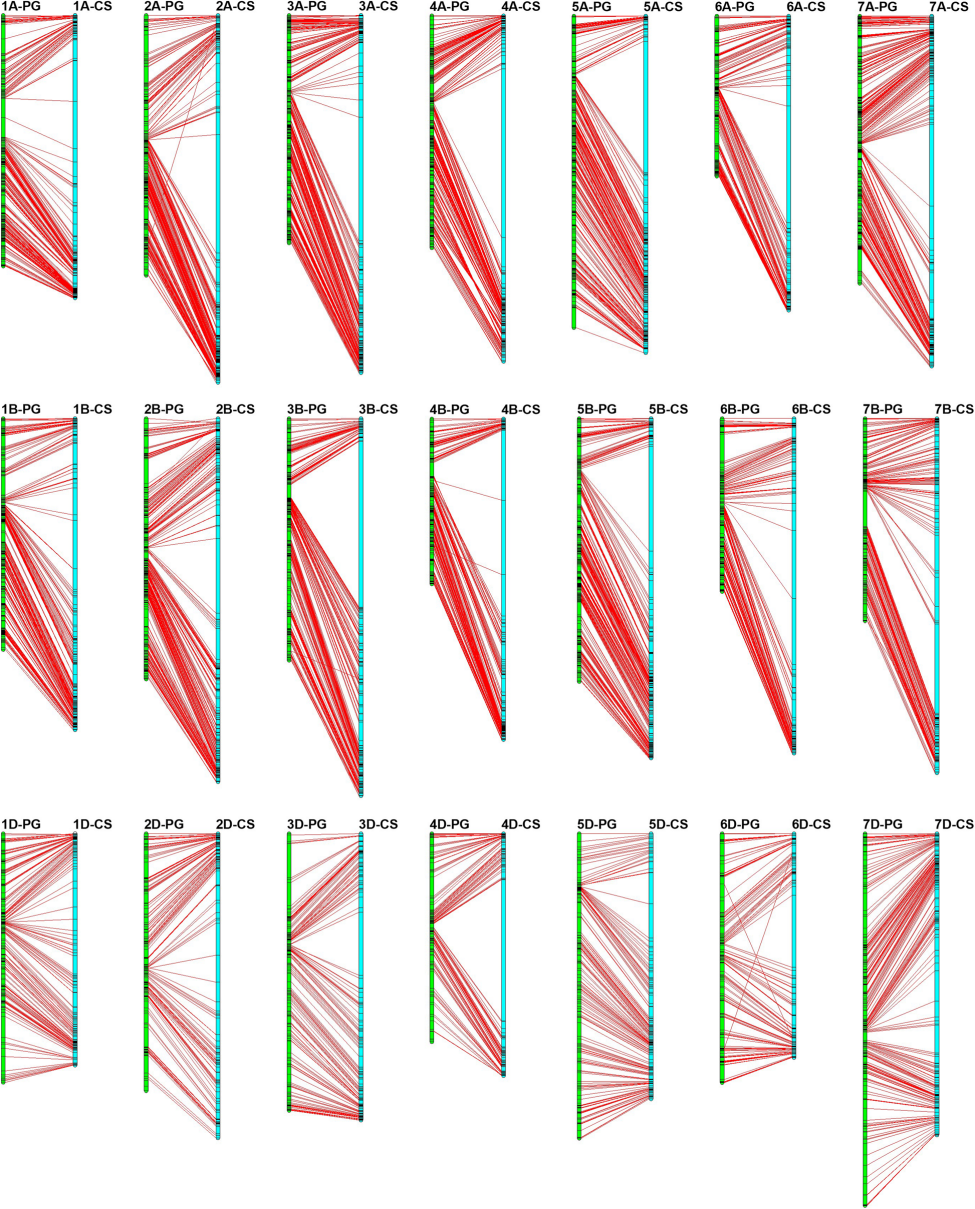
Collinearity between the genetic and physical positions (IWGSC RefSeq v1.0) for mapped SNPs in recombinant inbred line (RIL) population derived from PB3228/Gao8901 (PG-RIL) genetic map

Collinearity analysis indicated that the genetic map showed high collinearity with the wheat whole genome assembly IWGSC RefSeq v1.0, with the exception of chromosome 5BS, in which a segment inversion was detected (Fig. 2, Fig. S3). A total of 95.6% of the markers were located on the identical chromosomes between the PG-RIL linkage map and the physical map of IWGSC RefSeq v1.0 (Fig. S3).

### QTL analysis

A total of 79 QTLs (135 traits × environments) were detected, distributed on 18 chromosomes (Fig. S4, Table S2). The QTLs explained 0.5–33.0% of the phenotypic variation. Five environmentally stable QTLs were identified in at least three environments (Table S2). For GNS, 26 unconditional QTLs were detected (Fig. S4, Table S3). The PB3228- and G8901-derived alleles contributed to the additive effect for 15 and 11 QTLs, respectively. Two environmentally stable QTLs, *QGns-1A.1* and *QGns-4A.2*, were detected (Table 3). The QTL *QGns-4A.2* was significant in all the seven environments, with phenotypic variation explained (PVE) ranged from 9.6% to 13.1%; the RILs carrying the PB3228-allele had 3.21–4.39 more grains than that carrying the G8901-allele across the seven environments. The QTL *QGns-1A.1* was significant in three environments, with PVE ranged from 2.6% to 14.6%; the PB3228-allele can produce 2.89–7.26 more grains in the three environments (Table 3).

**Table 3:** Information of important QTLs related to grain number per spike (GNS) and their co-located QTLs for GNS-component traits

QTLs	Interval^a^	Environments	Unconditional and conditional QTLs for GNS												Unconditional QTLs for GNS component traits								
			GNS			GNS|GNSL			GNS|TSS			GNS|FSS			GNSL			TSS			FSS		
			LOD	PVE^b^	Add^c^	LOD	PVE	Add	LOD	PVE	Add	LOD	PVE	Add	LOD	PVE	Add	LOD	PVE	Add	LOD	PVE	Add
*QGNS-1A.1*	*0–7.5*	2012LC	3.0	6.3	4.37																		
		2015LC	3.3	2.6	2.89							4.5	6.1	2.98	4.6	6.5	0.17						
		2015SJZ	6.7	14.6	7.26				6.4	15.9	6.73	5.9	11.2	6.27									
*QGNS-3B.1*	*0–11.5*	2013LC							3.0	8.3	-2.81	3.3	8.3	-2.61									
		2015LC	4.5	3.5	-2.21				5.7	7.5	-2.61												
		2016LC										3.2	5.8	-1.44	4.1	8.5	-0.11						
*QGNS-3D.1*	*80.5–89.5*	2013LC																8.8	14.9	0.69			
		2014LC																6.1	10.4	0.44	3.2	7.3	0.36
		2015LC	3.7	2.9	2.01													11.3	19.1	0.52	8.8	19.7	0.52
		2015SJZ																6.9	10.7	0.47	3.1	3.4	0.38
		2016LC																15.7	21.5	0.65	13.4	12.9	0.55
*QGNS-4A.2*	*151.5–162.5*	2011LC	4.5	11.3	4.37																		
		2012LC	4.4	9.6	4.05																		
		2013LC	5.9	13.1	4.39													4.3	6.9	0.48			
		2014LC	5.9	10.0	3.82																		
		2015LC	12.1	10.4	3.83				6.3	8.5	2.80	4.6	6.0	1.98	5.4	7.7	0.13						
		2015SJZ	5.6	12.3	4.22				2.9	7.0	2.88	2.9	5.3	2.78				4.5	6.9	0.38			
		2016LC	12.3	10.8	3.21	5.5	1.1	1.53	7.7	4.3	2.48	4.7	8.8	1.80	2.7	5.6	0.09						
*QGNS-5A.1*	*33.5–37.5*	GNS14	4.4	7.2	-3.22																3.2	7.2	-0.36
		GNS15S	3.6	7.8	-3.33																		
*QGNS-7B.2*	*25.5–32.5*	2013LC	3.0	5.9	3.20																		
		2015LC							9.6	14.4	3.62	11.4	16.9	3.29	6.7	10.3	0.15						
		2016LC	11.1	9.7	3.68				3.8	2.0	1.71	5.8	11.2	2.01									
*QGNS-7D.2*	*103.5–109.5*	2011LC	4.7	12.3	4.62																		
		2015LC	14.8	13.5	4.42	4.6	10.3	1.73	4.3	5.6	2.29												

GNSL, TSS, FSS are the same as those in Table 1

^a^Marker interval means the interval of the LOD peak for QTLs

^b^Phenotypic variations explained by the QTLs

^c^The additive effects of the QTLs. Positive effect, increased effect contributed by PB3228; negative effect was contributed by G8901

Ten QTLs were identified for GNSL (Fig. S4, Table S2). The G8901-derived alleles conferred to the QTLs on chromosomes 2A, 3B, 4B and 7D, whereas the PB3228-derived alleles conferred to the other six QTLs on 1A, 3D, 4A, 5A, 5B and 7B. The QTLs *QGnsl-3D* and *QGnsl-4A* were detected in both the two investigated environments; and were correlated with *QGns-3D.2* and *QGns-4A.2*, respectively (Table 3). Six more QTLs *QGnsl-1A*, *QGnsl-3B*, *QGnsl-4B*, *QGnsl-5A*, *QGnsl-5B* and *QGnsl-7B* were also co-located with GNS-QTLs.

For the two SNS related traits TSS and FSS, 17 and 13 QTLs were detected, respectively (Fig. S4, Table S2). Four QTLs for TSS (*QTss-3D*, *QTss-4A*, *QTss-6A.2* and *QTss-7B.2*) and three for FSS (*QFss-3D.1*, *QFss-6A* and *QFss-7B.2*) were found to be significant in two or more environments (Table 3). Co-located environmentally stable QTLs for TSS and FSS were detected on chromosomes 3D (also co-located with *QGns-3D.1*), 6A, 6B and 7B (Table S2). The QTL *QTss-3D* was significant in five environments, with PVE ranged from 10.4% to 21.5%; while *QFss-3D.1* was significant in four environments, with PVE 3.4–19.7%. At this locus, the PB3228-derived allele improved TSS and FSS by 0.44–0.69 and 0.36–0.55, respectively. Co-locating of TSS/FSS-QTLs with GNS-QTLs was also found in 11 cases (Table S2).

The correlation coefficients between GNS|GNSL, GNS|TSS, GNS|FSS (conditional traits) and GNSL, TSS, FSS (unconditional traits) were shown in Table S4. A total of 22 conditional QTLs comprising 42 QTL × environments were detected for GNS after conditioned on GNSL, TSS or FSS (Fig. S4, Table S3). Among them, nine loci were also identified in unconditional analysis; five were co-located with unconditional QTLs for GNSL and one for both GNSL and TSS (Table 4). Thirteen QTLs were newly detected, among which five were co-located with unconditional QTLs for TSS or FSS. Different situations of QTL detection were found when using the conditional trait values of GNS conditioned on each of its components (Table S3). After conditioned on GNSL, six QTLs were identified for GNS, among which three were newly detected. Thirteen and twelve QTLs were detected by conditional values based on TSS and FSS, respectively, among which four and six were newly detected.

**Table 4:** The contribution information of the component traits to grain number per spike (GNS) at single QTL level

GNS QTLs	Unconditional and conditional QTLs							Contribution	
	GNS	GNS|GNSL	GNS|TSS	GNS|FSS	GNSL	TSS	FSS	GNSL	TSS/FSS
*QGNS-1A.1*	+		+	+	+			PB3228	
*QGNS-5B*	+		+	+	+			PB3228	
*QGNS-7B.2*	+		+	+	+			PB3228	
*QGNS-5A.2*	+	-	+	+	+			PB3228	G8901
*QGNS-3B.1*	-		-	-	-			G8901	
*QGNS-4B*	-		-		-			G8901	
*QGNS-4A.2*	+	+	+	+	+	+	+	PB3228	PB3228
*QGNS-7D.2*	+	+	+					PB3228	PB3228
*QGNS-3D.1*	+					+	+		PB3228
*QGNS-6B.3*	+						+		PB3228
*QGNS-7B.1*	+					+			PB3228
*QGNS-2A*	-					-			G8901
*QGNS-5A.1*	-						-		G8901
*QGNS-6B.2*	-					-	-		G8901
*QGNS-3D.2*	+				+		+	PB3228	PB3228

GNSL, TSS, FSS are the same as those in Table 1

### KASP marker and QTL validation

The SNPs closely linked to the two major QTLs *QGns-4A.2* (*AX-108966946* (chr4A:679986545) and *AX-109857541* (chr4A:681651241)) and *QGns-1A.1* (*AX-109901702* (chr1A:4072201) and *AX-109466381* (chr1A:8088981)) were converted to KASP markers. The markers were used to screen the PG-RIL and a diversity panel comprising of 141 accessions (Fig. S5). The genotyping result of the KASP markers for PG-RIL were almost the same as the Wheat 660 K SNP array; which mutually verified the accuracy of the two genotyping methods.

**Fig. 3:**
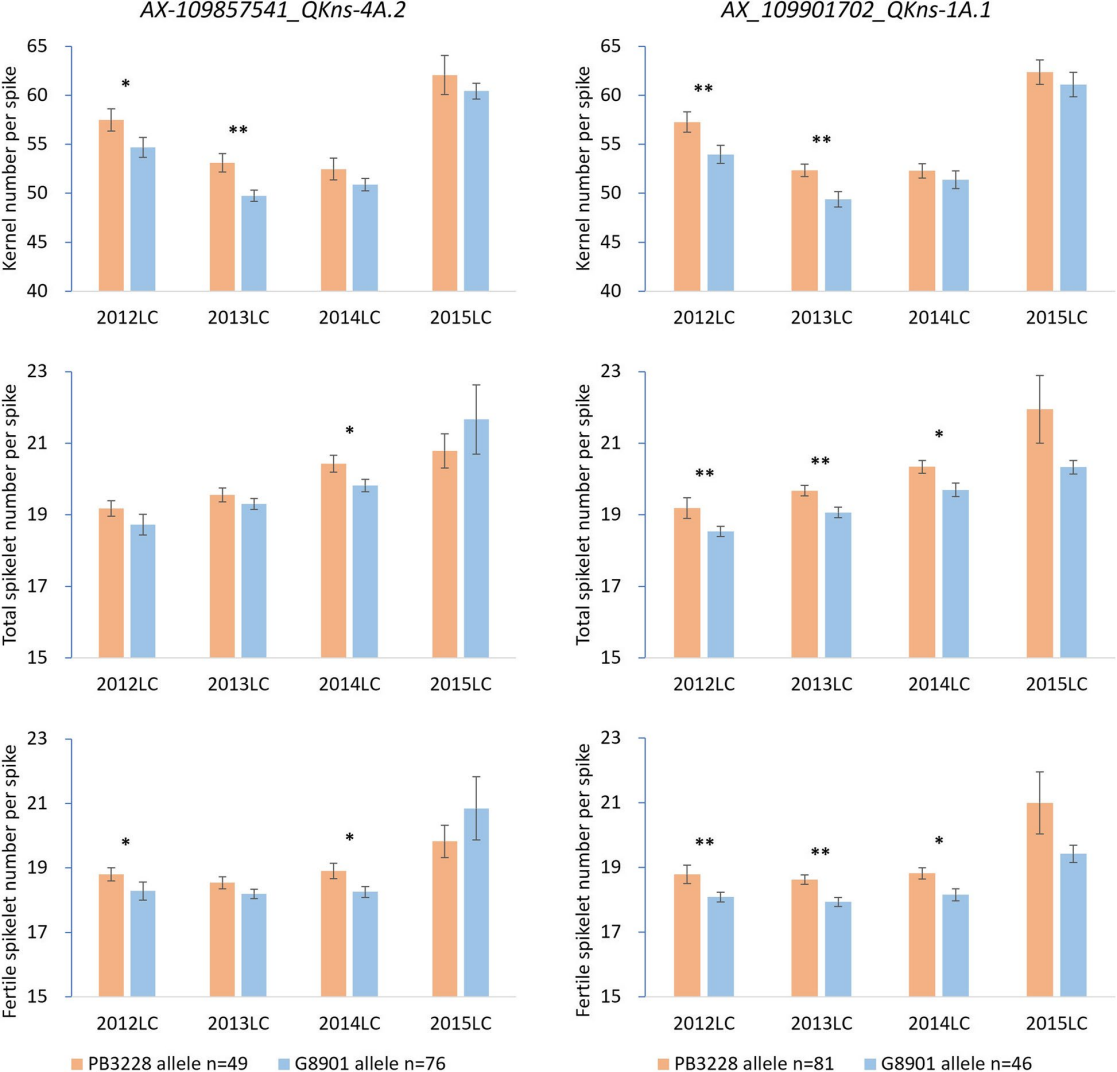
Phenotypic performance of different haplotypes for Grain number per spike (GNS), total- and fertile spikelet number per spike (TSS and FSS) in the diversity panel based on the kompetitive allele-specific PCR (KASP) markers *AX-109857541* (left) for *QGns-4A.2* and *AX-109901702* (right) for *QGns-1A.1*. * and ** represent the phenotype of opposite alleles was significantly different at *P* < 0.05 and* P* < 0.01 levels, respectively. Error bar stands for standard deviation

Two-tailed* t*-test was conducted based on the genotyping result of the KASP markers for both populations to verify the effects of the QTLs. Significant marker-trait correlations were found for all the four KASP markers. The two markers for *QGns-4A.2*, *AX-108966946* and *AX-109857541*, were correlated with GNS in all the seven environments (*P* < 0.001); and with GNSL and TSS/FSS in 20/24 of the trait × environments for PG-RIL (Table S5). They were also correlated with GNS and TSS/FSS in 6/24 of the trait × environments for the diversity panel (Fig. 3, Table S6). As for *QGns-1A.1*, *AX-109901702* and *AX-109466381* were correlated with GNS and GNSL in 3/14 and 2/4 of the trait × environments, respectively, but not with TSS/FSS in any environment for PG-RIL (Table S5). However, the two markers were correlated with GNS and TSS/FSS in 8/12 and 4/12 of the trait × environments, respectively, for the diversity panel (Fig. 3, Table S6). In the diversity panel, the PB3228 haplotype showed 1.24–3.39 and 0.62–3.30 more grains than G8901 haplotype for *QGns-4A.2* markers (*AX-108966946* and *AX-109857541*) and *QGns-1A.1* markers (*AX-109901702* and *AX-109466381*), respectively (Table S6).

### Prediction of candidate genes in the two key loci for GNS

In the present study, two important QTLs were detected for GNS. Among these, the QTL *QGns-4A.2* was identified in seven environments and the RILs carrying the PB3228-allele had 3.21–4.39 more grains than that carrying the G8901-allele. The physical confidence interval of *QGns-4A.2* was 2.6 Mb (679,072,862- 681,651,241 bp, RefSeq v1.0) and contained 28 high-confidence annotated genes (Table S8). Among these genes, *TraesCS4A02G407000* showed higher expression level in spike and grain [30] and may be the candidate gene for *QGns-4A.2*. The QTL *QGns-1A.1* was identified in three environments and the PB3228-allele can produce 2.89–7.26 more grains than the G8901-allele. The physical confidence interval of *QGns-1A.1* was 2.1 Mb (5,469,646- 7,536,121 bp, RefSeq v1.0) and contained 43 high-confidence annotated genes (Table S9). Among these genes, *TraesCS1A02G011300* showed much higher expression level in spike [30] and may be the candidate gene for *QGns-1A.1*. Further research is needed to firmly conclude the candidate genes.

## Discussion

### Comparison of the major QTLs with previous observations

One of the objectives of the present study was to identify constitutively-detected major QTLs for GNS and related traits. A total of five stable QTLs that were significant in at least three environments were detected. For GNS, the QTL *QGns-1A.1* was significant in three environments, with PVE ranged from 2.6% to 14.6%; the PB3228-allele can produce 2.89–7.26 more grains in the three environments. We located the QTL in the interval 5.5–7.5 Mb of chromosome 1A by BLAST-searching against the Chinese Spring reference genome sequence (RefSeq v1.0). The superior grain number character of wheat germplasm PB3228 was previously studied using a population of 237 F_2:3_ families from 3228/Jing 4839 [29] and a QTL for GNS and floret number per spikelet (FNS) was found in the interval *Xpsp3151-Xwmc24* on chromosome 1A. We located the two flanking markers at the positions 21.1 Mb and 27.3 Mb of 1A (RefSeq v1.0). Chen et al. [31] identified a QTL related to GNS near the position 458.4 Mb of 1A in the population derived from Pubing 3504/Jing 4839. Heidari et al. [20] detected a QTL for GNS near the position 4.1 Mb of 1A. Yuan et al. [32] found a QTL related to GNS near the position 344.7 Mb of 1A using a double haploid (DH) populations derived from Huapei 3/Yumai 57. Using the same population, Deng et al. [33] identified a QTL related to GNS near the position 159.5 Mb of 1A. Xu et al. [8] detected a QTL for GNS near the position 280.6 Mb of 1A in the population derived from Xiaoyan 54/Jing 411. Zhai et al. [34] identified two QTLs related to GNS near the position 320.2 Mb and 496.3 Mb of 1A using the population derived from Yumai 8679/Jing 411. By comparison of the positions the two QTLs, we found that *QGns-1A.1* detected in this study was different from the QTL detected in the previous studies and might be a novel QTL.

In this study, *QGns-4A.2* was significant for GNS in seven environments in the interval *AX-109857541*-*AX-110540586*-*AX-111486749*-*AX-108837287*. The interval was further mapped to a 2.58 Mb physical interval (4A: 679072862–681651241) based on the IWGSC RefSeq v1.0. The common SNP marker *AX-110540586* (4A: 680398704) was also found significantly linked to QTLs for GNS in “Xiaoyan 54/Jing411” RIL population [35]. In an association analysis using the Wheat 90 K SNP array, *RAC875_c59673_500* (4A: 681670895) was found to be associated with GNS [36]. A major QTL for GNS was reported to be significant in ten environments in the interval *AX-109844107*-*AX-110540586*, which had an overlapping physical interval (4A: 680398739–683638403) as our result [37]. The results indicated that *QGns-4A.2* detected in the present study was a stable QTL that could be identified in different environments and different trails, which represented a valuable target for marker-assisted selection to enhance GNS in wheat breeding.

Besides, the environmentally stable QTL *QTss-3D* was significant for TSS in five environments, explaining 10.4–21.5% of the phenotypic variations. The QTL was also detected for FSS in four environments (*QFss-3D.1*), explaining 3.4–19.7% of the phenotypic variations. The QTL was located in the interval *AX-109499958-AX-109516184-AX-108807742-AX-111064903* and mapped to mapped to the physical interval 65.8–124.0 Mb based on the IWGSC RefSeq v1.0. The gene *WAG2-3D* was reported to be involved in wheat spikelet development and located at the position 100.9 Mb on chromosome 3D [38]. This gene may be one of the candidate genes of *QTss-3D/QFss-3D.1* and need further analysis.

### How do PB3228 alleles contribute to GNS at individual QTL level?

Wild relatives of wheat are a reservoir of genetic diversity for improving wheat productivity. Introgression from wild relatives have a great potential to broaden the availability of beneficial allelic diversity for wheat improvement in breeding programs. *Agropyron cristatum* (2n = 4x = 28, PPPP) is a wild relative of common wheat and confers several desirable agronomic traits to wheat, such as high grain number per spike [39]. Unfortunately, many translocation lines have large and complex chromosome segments, leading to questionable value in wheat breeding program. In this study, the wheat germplasm PB3228 was obtained through a cross between the wheat cultivar “Fukuhokomugi” and the tetraploid *A. cristatum* accession Z559. Accession Z559 possesses several favorable traits, including a superior spike with a high grain number [40]. During the 1990s, accession Z559 was crossed with Fukuhokomugi and line 4844 was chosen based on the significant spike size observed in the progeny lines [40]. The characterization of chromosome addition and substitution lines derived from line 4844 through in situ hybridization, SSR, and gliadin analyses revealed that the increased numbers of florets and kernels within a spike of line 4844 were genetically controlled by gene(s) located on chromosome 6P of *A. cristatum* [41]. After several rounds of selection, a genetically stable derivative exhibiting elite characteristics was obtained from the descendants of the wheat-*A. cristatum* chromosome addition line 4844–12 (2n = 44). This derivative, named PB3228, showed exceptional yield traits, including a high grain number and an impressive number of superior florets per spike. We developed a RIL population derived from the cross of PB3228 / Gao 8901 and constructed a high-density genetic linkage map to identify stable QTLs for grain number per spike (GNS) from PB3228, which will contribute favorable QTLs for MAS and fine-mapping and facilitate yield improvement in wheat breeding.

Furthermore, we carried out conditional analysis to identify QTLs for GNS based on trait values conditioned on each of its component traits. The results indicated that GNSL and FSS/TSS were both important for GNS; GNSL contributed a little more than FSS/TSS at the genetic background of PG-RIL population, which was consistent with the results of correlation analysis.

The unconditional QTL *QGns-1A.1* was identified in three environments and the PB3228-allele can produce 2.89–7.26 more grains than the G8901-allele. The QTL was also found when conditioned on TSS or FSS but failed to be detected when conditioned on GNSL (Table 4). In the same interval, a QTL for GNSL was detected, but none for TSS/FSS. Therefore, the PB3228-derived allele contributed to *QGns-1A.1* by improving GNSL. The situation of *QGns-5B* and *QGns-7B.2* were the same as *QGns-1A.1*. *QGns-5A.2* was a little different. A conditional QTL conferred by G8901 was also detected after conditioned on GNSL. Besides the influence of GNSL conferred by PB3228, G8901 may also had some effect on this QTL.

The unconditional QTL *QGns-4A.2* was identified in seven environments and the RILs carrying the PB3228-allele had 3.21–4.39 more grains than that carrying the G8901-allele. The QTL was also detected after conditioned on either GNSL (with sharply dropped PVE) or TSS/FSS (with slightly dropped PVE). In the same interval, QTLs for GNSL (*QGnsl-4A*) and TSS (*QTss-4A*) were also detected (Table 4 and Table S3). This locus may be controlled by both components, and GNSL had a larger effect than TSS/FSS. The PB3228-derived allele contributed to GNS by improving both GNSL and TSS/FSS. *QGns-7D.2* was similar to *QGns-4A.2*, but no QTL was found for GNSL or TSS/FSS. Considering the PVE reduction for conditional analysis, the PB3228-derived allele had positive effect on GNS by improving GNSL and TSS simultaneously, and TSS had a greater effect than GNSL.

The QTLs *QGns-3B.1* and *QGns-4B* were also detected when GNS was conditioned on TSS or FSS and failed on GNSL, but with negative additive effect (Table 4). QTLs for GNSL with negative additive effect were also identified, indicated that G8901-derived alleles for *QGns-3B.1* and *QGns-4B* have positive effect on GNS by improving GNSL.

The relationships between GNS and its components were also predicted by QTL clustering. The three QTLs *QGns-3D.1*, *QGns-6B.3* and *QGns-7B.1* were co-located with TSS/FSS QTLs with positive additive effect. PB3228-derived alleles may contribute to GNS by improving TSS/FSS (Table 4). Besides, there were also three QTLs (*QGns-2A*, *QGns-5A.1* and *QGns-6A.2*) that were influenced by TSS/FSS and conferred by G8901-derived alleles. *QGns-3D.2* was clustered with QTLs for both GNSL and FSS with positive additive effect. The PB3228-derived allele may have positive effect on GNS by improving both GNSL and FSS (Table 4).

Using a high-density genetic linkage map, unconditional and conditional QTL analysis dissected the contribution of GNSL and SNS to GNS at individual QTL level. In PG-RIL, PB3228 contributed to most of the GNS-QTLs by improving either or both of GNSL and SNS. The environmental-stable QTLs such as *QGns-4A.2*, *QGns-1A.1*, *QGns-3D.1* and *QGns-7B.2* were more preferable for further study. The KASP markers for *QGns-4A.2* and *QGns-1A.1* can be used in MAS for wheat high-yield improvement.

### The advantage of SNP markers and high-density genetic map

Over the past two decades, a number of linkage maps of wheat have been reported and successfully applied to identify QTLs for various wheat traits [8, 42–46]. The genetic maps were mainly composed of PCR-based molecular markers such as SSR, EST-SSR, RAPD and AFLP, etc. [2, 47–49]. However, most of the linkage maps were with restricted marker density and the mapped QTLs were within relatively large confidence intervals [13]. This greatly hindered the applications of the detected QTLs in wheat breeding programs and further fine mapping of the QTLs. On the other hand, researchers attempted to compare the results of different trials using diverse genetic populations and different linkage maps to confirm their findings and to identify stable QTLs that warrant further study [4, 8–10, 13, 42, 50]. However, most of the genetic maps differed greatly in molecular markers; and the PCR-based markers such as SSR were short of sequence information and difficult to be located precisely in the reference genome sequence. Therefore, identifying loci or genes with the same type of marker is a promising way for researchers to share their achievement more efficiently.

The SNP marker with accurate physical position which is generally more abundant, stable and efficient is the best alternate choice. SNP arrays are flexible in terms of sample and data point number customization, which contributes to its high-density scanning and high robust call rates compared to PCR-based markers. Using the high-throughput SNP arrays, consensus genetic maps have been released [46, 51–53]. High-density linkage maps based on bi-parental populations were also reported using the SNP arrays, such as the wheat 9 K, 90 K, 55 K and 660 K arrays [11, 13, 16, 37, 54, 55]. The maps were used in QTL mapping for GNS, grain size, spike-related and processing quality traits in bread wheat. The information of tightly-linked SNPs facilitated the comparative analysis and candidate gene prediction of the QTLs [13, 37]. The high-density linkage map also allowed the possibility of comparing its collinearity with the physical map. The high collinearity of our genetic map with the physical map indicated no obvious structural variation between PB3228 and Chinese Spring. Sun et al. [56] evaluated seven types of wheat SNP arrays and found that the Wheat 660 K SNP array may be the best choice for genetic analysis in wheat.

## Materials and methods

### Plant materials

The population used for constructing the map and performing QTL mapping consisted of 176 F_2:9_ RILs, which were derived through single seed descent from a cross between PB3228 and G8901. PB3228 was a germplasm with superior features in spike morphological traits, such as large spike size and superior grain number (Fig. S1) [3, 29]. On the other hand, G8901 was a strong gluten line, with a grain number lower than PB3228, but higher than that of most cultivated varieties. To validate the effects of SNPs linked to major QTLs, a diversity panel consisting of 141 wheat accessions was utilized.

### Field trials

During the growing seasons from 2011–2012 (2011LC) to 2016–2017 (2016LC), six field trials were conducted at the Luancheng Agro-ecosystem Station, the Chinese Academy of Sciences. The coordinates of the Station are 37° 53′ 15″ N, 114° 40′ 47″ E, with an elevation of 50 m. During the 2015–2016 (2015SJZ) growing season, one trail was conducted at the greenhouse of the Center for Agricultural Resources Research, Institute of Genetics and Developmental Biology, Chinese Academy of Sciences located at Shijiazhuang. Hereafter, “2011LC”, “2012LC”, “2013LC”, “2014LC”, “2015LC”, “2015SJZ” and “2016LC” represent the seven environments, respectively.

The four trials conducted in the environments of 2013LC, 2014LC, 2015LC, and 2016LC followed a completely randomized design (CRD) with three replications. The three replications were designated as the main plots, while the 176 RILs and their parents served as subplots.

Seeds were planted in early October, and plants were harvested in mid-June once they reached physiological maturity. Each subplot covered an area of 1.5 m^2^ and contained four rows, with each row measuring 1.5 m in length and spaced 0.25 m apart. Twenty seeds were planted in each row. Adequate watering was provided for all fields, and local standard protocols for field trial management were followed. In the case of 2011LC, 2012LC and 2015SJZ, only a single row measuring 1.5 m in length was planted with 20 seeds for each line.

The diversity panel was cultivated across four environments 2012LC, 2013LC, 2014LC, and 2015LC. The experimental design and phenotyping methods used in these trails were described as in Ma et al. [36].

### Evaluation of traits

For the five environments (2013LC, 2014LC, 2015LC, 2015SJZ, and 2016LC), a sample of ten representative plants was taken from each plot. In case of for 2013LC, 2014LC, 2015LC, and 2016LC, the ten representative plants were chosen from the middle of the two internal rows. The traits GNS, TSS, and FSS were calculated by averaging the values observed from the main spikes of these ten plants. For 2015LC and 2016LC, the maximum GNSL of the main spikes was documented. For 2011LC and 2012LC, the GNS of the main spike from a randomly selected plant was recorded.

### Genotyping and genetic map construction

The parental lines, along with 176 PG-RILs were genotyped using the Wheat 660 K SNP array (https://wheat.pw.usda.gov/ggpages/topics/Wheat660_SNP_array_developed_by_CAAS.pdf) at China Golden Marker Corporation (Beijing, China; http://cgmb.com.cn/). In addition to this, the RILs were screened using 40 SSR markers, two KASP markers, and two markers identifying glutenin subunits (Table S7). A morphological marker for awn length was also used during the construction of genetic map. SNPs that met the criteria of having a Call Rate > 97%, Heterozygote Rate < 20%, and Minor Allele Frequency > 5% were selected for map construction. To eliminate redundant markers, the BIN function in QTL IciMapping V4.1 (http://www.isbreeding.net/) was employed for binning purposes [57]. For each bin, only one marker with the smallest missing rate was retained. The MAP function was used to construct linkage map for the PG-RIL population. SNPs were grouped together with a LOD score of 3.5 and then ordered using the nnTwoOpt function. Rippling function was carried out with a SARF criterion and a window size of 5. The confirmation of the 21 chromosomes and marker order was done based on the physical position of most SNPs in the IWGSC RefSeq v1.0 [26]. The genetic map was drawn using MapChart 2.2 [58].

### Data analysis and QTL mapping

The data was analyzed using the AOV function in QTL ICIMapping V4.1 to perform Analysis of Variance (ANOVA). The broad-sense heritability (*h*_*B*_^*2*^) was determined by calculating V_G_/V_P_; where V_G_ represents the genetic variance and V_P_ represents the phenotypic variance. The environments were regarded as replications, and the genotype × environment interaction was considered as the error term [8, 50].

QTL mapping was carried out using the constructed linkage map of PG-RIL. The BIP function, based on inclusive composite interval mapping (ICIM), in QTL IciMapping v4.1 was utilized for this purpose. Additionally, conditional analysis was conducted to investigate the effects of the component traits on the expression of QTL for GNS. The conditional phenotypic values (y_(GNS|GNSL)_, y_(GNS|TSS)_ and y_(GNS|FSS)_) represent the net genetic variation of trait values for GNS that is independent of GNSL, TSS or FSS. These values were evaluated using QGAStation 1.0 (http://ibi.zju.edu.cn/software/qga/). The raw data from each environment were organized as follows: the first column indicated the block (replications), the second column represented the genotype (176 RILs), and the subsequent columns contained the trait data. Conditional genetic analysis was performed by considering “Conditional on Final Stage” for traits in each individal environment. Both the observed and conditional phenotypic values were utilized for QTL analysis, with the identified QTLs categorized as unconditional and conditional QTLs, respectively. A LOD threshold of 2.5 was set to declare a significant QTL. In cases where QTLs had overlapping confidence intervals, they were considered equivalent. Finally, all QTLs were named according to Fan et al. [59].

### Conversion of SNPs to KASP markers

Conversion of SNPs to KASP markers was conducted for the SNPs closely linked to the two major QTLs *QGNS-4A.2* and *QGNS-1A.1* (Table S7). These KASP markers were subsequently utilized in the screening of the PG-RIL population and the diversity panel. The PCR were performed in a reaction volume of 6 μl, consisting of 0.0825 μl KASP primer mix, 3 μl 2 × KASP Master Mix (LGC Genomics, UK), and 3 μl genomic DNA at a concentration of 20 ng/μl. The fluorescence emitted during the reaction was detected using the BIO-RAD CFX real-time PCR system, and the obtained results were analyzed with Bio-Rad CFX Manager 3.1 software.

### Supplementary Information

**Additional file 1: Table S1.** Marker sequences for map construction and marker-assisted selection.

**Additional file 2: Table S2.** Analysis of variance (ANOVA), the heritability (hB2), and average phenotypic performance for the investigated traits of the recombinant inbred lines (RILs) and their parents in seven environments.

**Additional file 3: Table S3.** Summary of unconditional and conditional QTLs for GNS and its component traits.

**Additional file 4: Table S4.** The correlation coefficients between GNS|GNSL, GNS|TSS, GNS|FSS (conditional traits) and GNSL, TSS, FSS (unconditional traits).

**Additional file 5: Table S5.** Unconditional and conditional QTLs for GNS and their co-located QTLs for GNS-component traits.

**Additional file 6: Table S6.** Phenotypic performance of different haplotypes in PG-RIL population based on the KASP markers for QGns-4A.2 and QGns-1A.1.

**Additional file 7: Table S7.** Phenotypic performance of different haplotypes in the diversity panel based on the KASP markers for QGns-4A.2 and QGns-1A.1.

**Additional file 8: Table S8.** High-confidence annotated genes located in the confidence interval of *QGns-4A.2*.

**Additional file 9: Table S9.** High-confidence annotated genes located in the confidence interval of *QGns-1A.1*.

**Additional file 10: Fig. S1.** Morphology of plant performance (A), spikes (B) and spikelets (C) of the parental lines PB3228 (left) and G8901 (right) grown in Luancheng (2016–2017 growing season). **Fig. S2.** Frequency distribution of GNS and its component traits for PG-RILs determined in various environments. **Fig. S3.** Comparision between the genetic and physical location (IWGSC RefSeq v1.0) for mapped SNPs in PG-RIL genetic map. **Fig. S4.** Allelic segregation of KASP markers *AX-108966946* and *AX-109857541* for *QGns4A.2*, and *AX-109901702* and *AX-109466381* for *QGns-1A.1* in AM141 population (left) and PG-RIL population (right). **Fig. S5.** Allelic segregation of KASP markers AX-108966946 and AX-109857541 for QGns-4A.2, and AX-109901702 and AX-109466381 for QGns-1A.1 in the diversity panel (left) and PG-RIL population (right). Varieties colored blue have the HEX-type allele, varieties colored red have the FAM-type allele, varieties colored green are heterozygote that have the two types of alleles, black dots represent the NTC (non-template control).

## Data Availability

The original contributions presented in the study are included in the article/Supplementary Material, further inquiries can be directed to the corresponding authors.

## Abbreviations

FSS: Fertile spikelet number per spike
GNS: Grain number per spike
GNSL: Grain number of spikelet
KASP: Kompetitive allele-specific PCR
QTL: Quantitative trait locus
RIL: Recombinant inbred line
SNP: Single nucleotide polymorphisms
SNS: Spikelet number per spike
SSS: Sterile spikelet number per spike
TSS: Total spikelet number per spike
