# Supplementary material for: Unraveling the genetic basis of grain number-related traits in a wheat-Agropyron cristatum introgressed line through high-resolution linkage mapping
Source: BMC Plant Biol. 2023 Nov 15;23:563. doi: 10.1186/s12870-023-04547-7 (PMC10647127; doi:10.1186/s12870-023-04547-7)
Supplement: Supplementary file 10 — Additional file 10: Fig. S1. Morphology of plant performance (A), spikes (B) and spikelets (C) of the parental lines PB3228 (left) and G8901 (right) grown in Luancheng (2016–2017 growing season). Fig. S2. Frequency distribution of GNS and its component traits for PG-RILs determined in various environments. Fig. S3. Comparision between the genetic and physical location (IWGSC RefSeq v1.0) for mapped SNPs in PG-RIL genetic map. Fig. S4. Allelic segregation of KASP markers AX-108966946 and AX-109857541 for QGns4A.2, and AX-109901702 and AX-109466381 for QGns-1A.1 in AM141 population (left) and PG-RIL population (right). Fig. S5. Allelic segregation of KASP markers AX-108966946 and AX-109857541 for QGns-4A.2, and AX-109901702 and AX-109466381 for QGns-1A.1 in the diversity panel (left) and PG-RIL population (right). Varieties colored blue have the HEX-type allele, varieties colored red have the FAM-type allele, varieties colored green are heterozygote that have the two types of alleles, black dots represent the NTC (non-template control). [file 12870_2023_4547_MOESM10_ESM.docx]

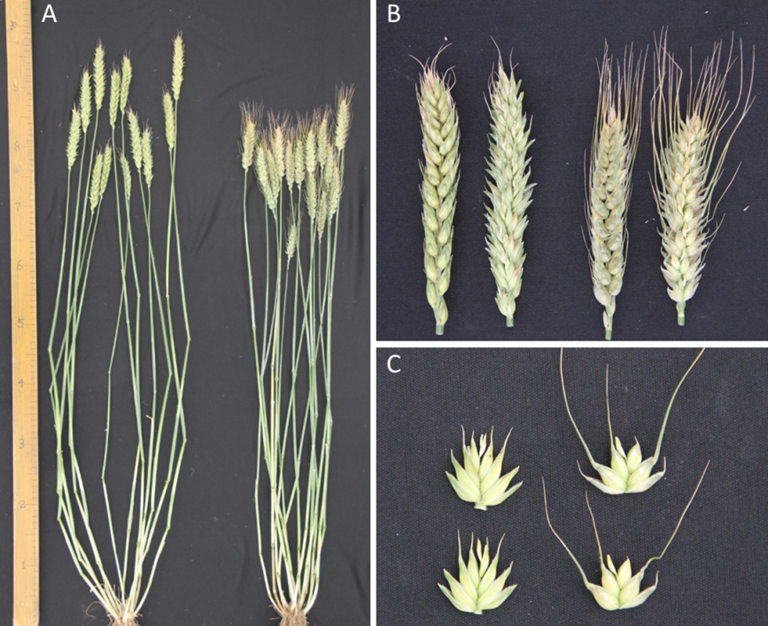


**Fig. S1** Morphology of plant performance (A), spikes (B) and spikelets (C) of the parental lines PB3228 (left) and G8901 (right) grown in Luancheng (2016–2017 growing season).

**
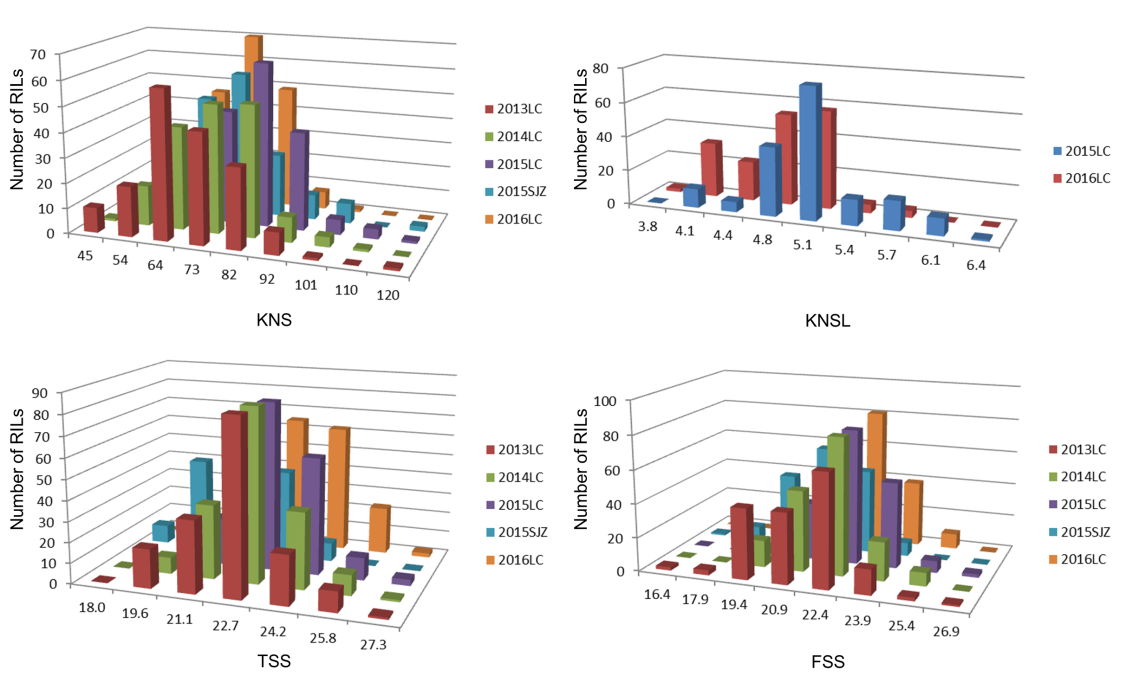
**

**Fig. S2** Frequency distribution of GNS and its component traits for PG-RILs determined in various environments.


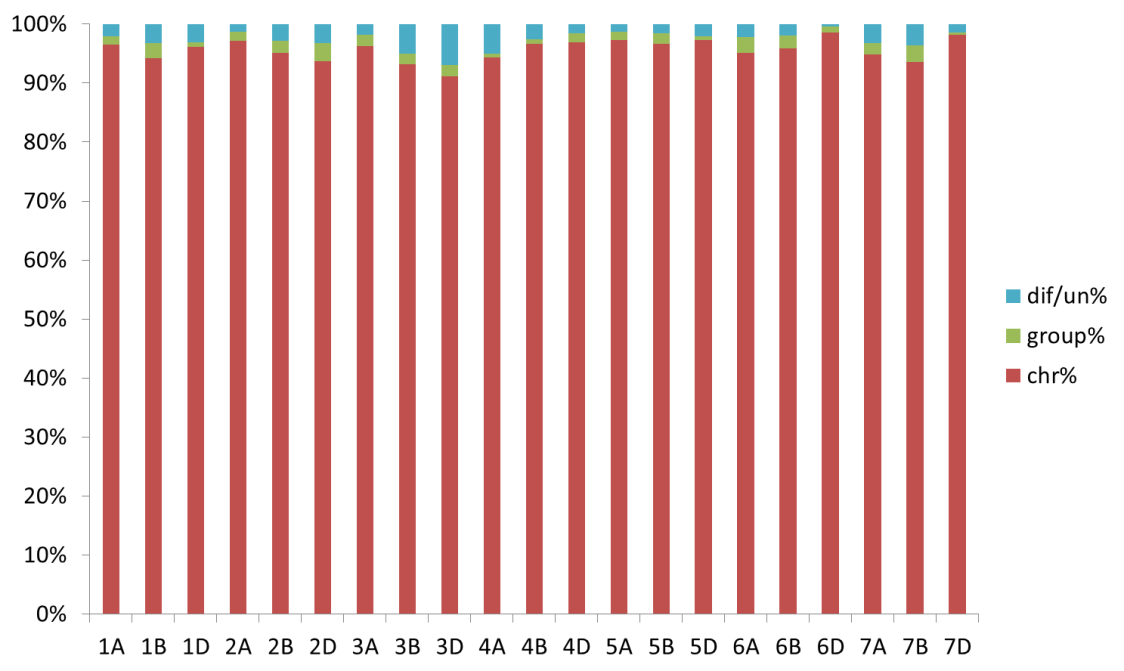
**Fig. S3** Comparision between the genetic and physical location (IWGSC RefSeq v1.0) for mapped SNPs in PG-RIL genetic map. The length of histogram in red color indicates the percentage of markers with coincident physical and genetic positions (identical chromosome), that in green color indicates the percentage of markers that are mapped to homoeologous chromosomes compared with the physical positions, that in blue color indicates the percentage of markers with inconsistent physical and genetic positions (in disorder) or with unknown physical position.


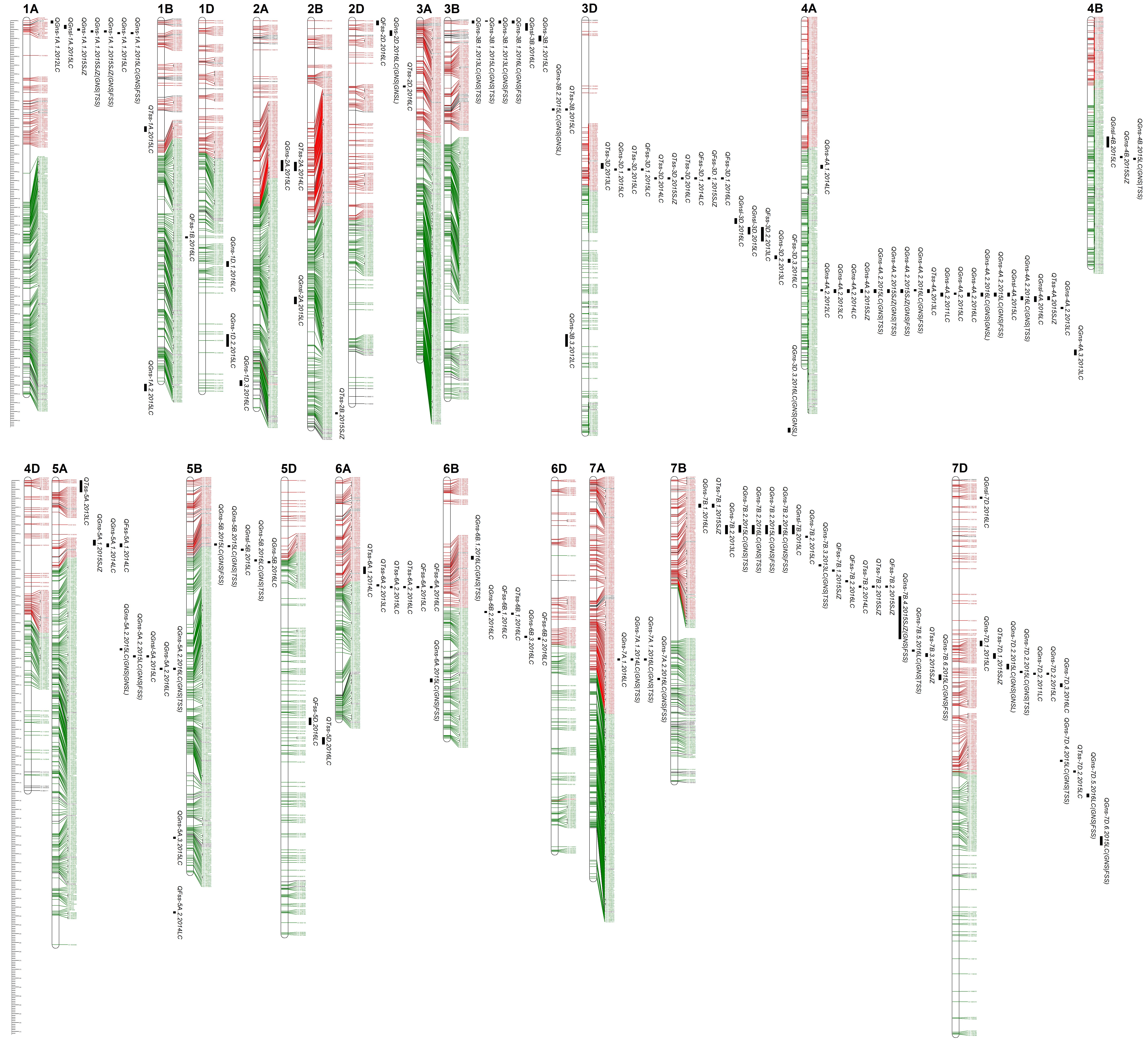


**Fig. S4** Locations of QTLs detected for GNS and its component traits is based on PG-RIL by unconditional and conditional analysis


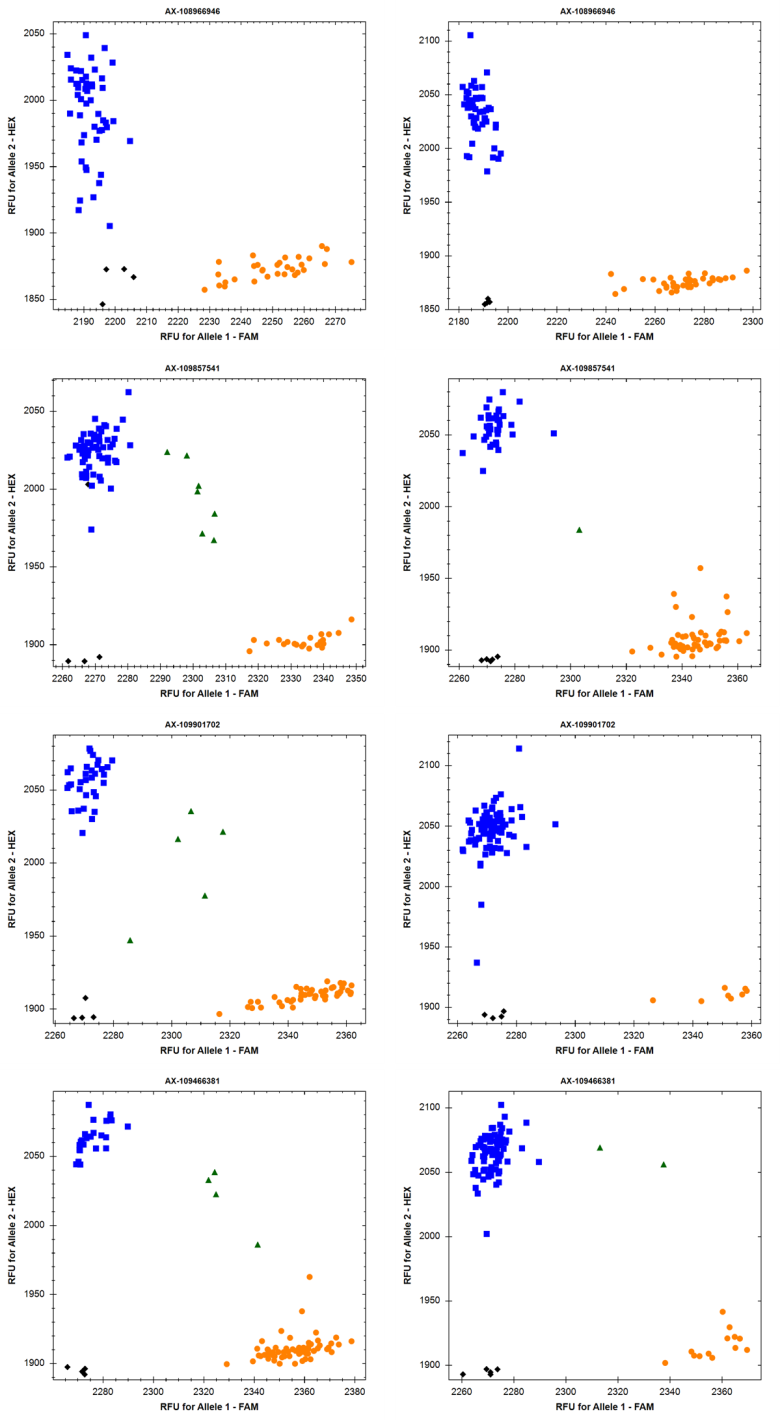


**Fig. S5** Allelic segregation of KASP markers *AX-108966946* and *AX-109857541* for *QGns-4A.2*, and *AX-109901702* and *AX-109466381* for *QGns-1A.1* in the diversity panel (left) and PG-RIL population (right). Varieties colored blue have the HEX-type allele, varieties colored red have the FAM-type allele, varieties colored green are heterozygote that have the two types of alleles, black dots represent the NTC (non-template control).
